# P-1069. Patterns and Prevalence of Carbapenemase-Producing Organisms Identified in Nebraska Healthcare Facilities Following Whole Genome Sequencing (2019-2024)

**DOI:** 10.1093/ofid/ofaf695.1264

**Published:** 2026-01-11

**Authors:** Kathryn C Burbach, Ishrat Kamal-Ahmed, Muhammad Salman Ashraf, Amy Roden, Peter C Iwen, Lacey Pavlovsky, Juan M Teran Plasencia

**Affiliations:** Nebraska Department of Health and Human Services, Omaha, NE; Division of Public Health, Nebraska Department of Health and Human Services, Lincoln, Nebraska; University of Nebraska Medical Center; Nebraska Public Health Laboratory, Omaha, Nebraska; Nebraska Public Health Laboratory, Omaha, Nebraska; Nebraska Department of Health and Human Services, Omaha, NE; University of Nebraska Medical Center/Division of Infectious Diseases, Omaha, NE

## Abstract

**Background:**

Carbapenemase-producing organisms (CPOs) pose threats to public health, are associated with worse clinical outcomes and heightened risk for transmission. Whole Genome Sequencing (WGS) offers enhanced outbreak detection compared to traditional methods. We evaluate the use of WGS for the detection and characterization of CPOs in Nebraska.

**Figure:**
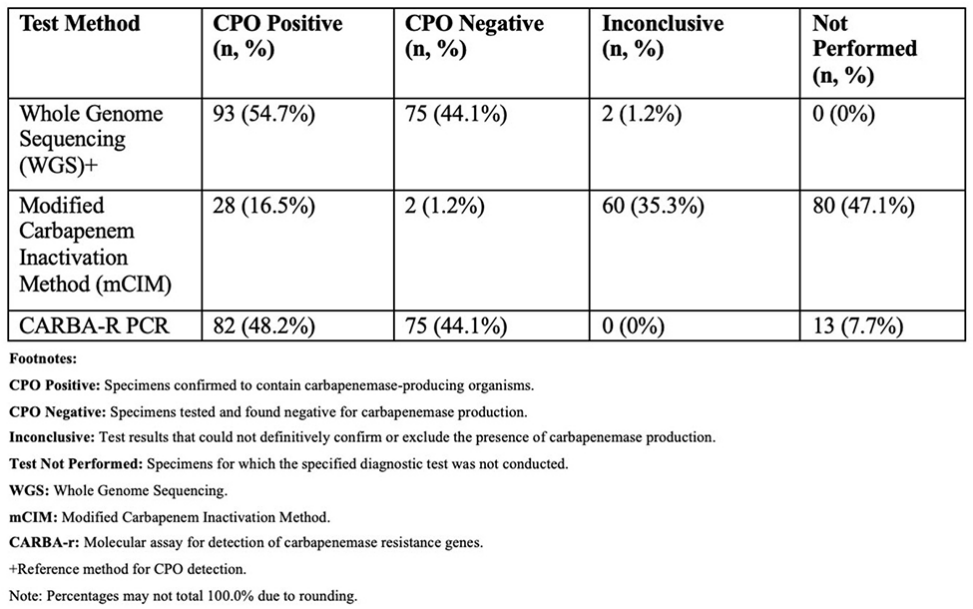
Detection of Carbapenemase-Producing Organisms (CPO) Among 170 Carbapenem-Resistant Isolates by Three Test Methods, Nebraska, 2019-2024

**Figure:**
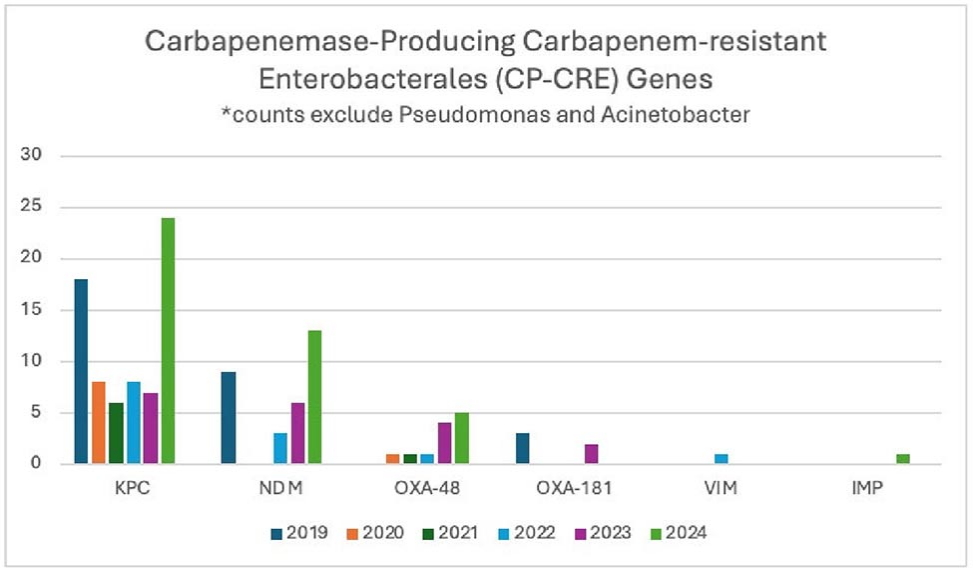

**Methods:**

This is a retrospective review of clinical and screening isolates submitted to the Nebraska Public Health Laboratory (NPHL) from 2019-2024. Carbapenem-resistant *Acinetobacter* spp, *Enterobacterales* (minor exceptions), and *Pseudomonas* (cefepime resistant) along with other gram-negative pathogens are frequently submitted by clinical laboratories for additional testing, including mCIM and/or PCR for carbapenemase genes. Positive and indeterminate results underwent WGS using the Clear Labs Clear Dx platform with the Microbial Surveillance 2.0 kit and the onboard Illumina iSeq system. Data was processed using the CDC’s Phoenix pipeline and cross-referenced with the NCBI AMR Catalog. Descriptive statistics were conducted using SAS 9.4.

**Results:**

A total of 170 isolates were included, 90 underwent mCIM testing (31% positive, 66.6% inconclusive), 157 underwent Carba-R (52.2% positive), and all underwent WGS, of which 93 (54.7%) harbored a carbapenemase gene. *E.coli* (31.18%) and *K.pneumoniae* (15.05%) were the most prevalent organisms identified among CPOs. Organism-gene pairings with *E.coli*-NDM (18.28%) and *C.freundii*-KPC (8.6%) were most common among all isolates. The most frequently identified carbapenemase genes were KPC (36.56%), NDM (33.33%), OXA-48-like (22.58%), VIM (1.08%), and IMP (1.08%). Of 26 isolates where any OXA carbapenemase allele was identified, 80.77% belonged to the OXA-48-like gene family. Among 60 inconclusive mCIM isolates, only one harbored a carbapenemase gene. Among WGS isolates without a carbapenemase gene, all but one harbored an ampC or related class C beta-lactamase. Carba-R missed 8 CPOs detected by WGS, mostly in *Acinetobacter* spp.

**Conclusion:**

WGS improved detection and characterization of CPOs in Nebraska, offering higher discriminatory power than traditional methods. Further research is needed to optimize its use for outbreak detection and guide infection control efforts.

**Disclosures:**

Muhammad Salman Ashraf, MBBS, Merck & Co. Inc: Grant/Research Support

